# Recycled Utilization of a Nanoporous Au Electrode for Reduced Fabrication Cost of Perovskite Solar Cells

**DOI:** 10.1002/advs.201902474

**Published:** 2020-01-30

**Authors:** Fengjiu Yang, Jinzhe Liu, Zheng Lu, Pengfei Dai, Tomoya Nakamura, Shenghao Wang, Luyang Chen, Atsushi Wakamiya, Kazunari Matsuda

**Affiliations:** ^1^ Institute of Advanced Energy Kyoto University Uji Kyoto 611‐0011 Japan; ^2^ School of Materials Science and Engineering East China University of Science and Technology Shanghai 200237 China; ^3^ Materials Genome Institute Shanghai University Shanghai 200444 China; ^4^ Institute for Chemical Research Kyoto University Uji Kyoto 611‐0011 Japan

**Keywords:** bending durability, nanoporous Au films, perovskite solar cells, recycled utilization, reduce fabrication costs

## Abstract

Perovskite solar cells (PSCs) using metal electrodes have been regarded as promising candidates for next‐generation photovoltaic devices because of their high efficiency, low fabrication temperature, and low cost potential. However, the complicated and rigorous thermal deposition process of metal contact electrodes remains a challenging issue for reducing the energy pay‐back period in commercial PSCs, as the ubiquitous one‐time use of a contact electrode wastes limited resources and pollutes the environment. Here, a nanoporous Au film electrode fabricated by a simple dry transfer process is introduced to replace the thermally evaporated Au electrode in PSCs. A high power conversion efficiency (PCE) of 19.0% is demonstrated in PSCs with the nanoporous Au film electrode. Moreover, the electrode is recycled more than 12 times to realize a further reduced fabrication cost of PSCs and noble metal materials consumption and to prevent environmental pollution. When the nanoporous Au electrode is applied to flexible PSCs, a PCE of 17.3% and superior bending durability of ≈98.5% after 1000 cycles of harsh bending tests are achieved. The nanoscale pores and the capability of the porous structure to impede crack generation and propagation enable the nanoporous Au electrode to be recycled and result in excellent bending durability.

## Graphical abstract

Nanoporous Au film is successfully introduced into perovskite solar cells to replace the typical thermal deposition of metal electrode with a high efficiency of 19.0% on rigid substrate and sustains an excellent bending durability of 98.5% even after 1000 cycles testing on a flexible device, while its facile and recycled utilization significantly reduces the device fabrication cost, noble metal consuming, and environmental pollution.

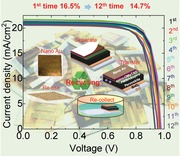

## 1 Introduction

Organic/inorganic hybrid perovskites are the most promising materials for use in solar cells as light absorption layers because of their superior properties, including high carrier mobility,^1^ long carrier diffusion,^2^ large light absorption coefficient,^3^ tunable bandgap,^4^, ^5^, ^6^ low exciton binding energy,^2^ and potential for low‐temperature fabrication process.^7^ Organic/inorganic hybrid perovskite solar cells (PSCs) have witnessed an explosive development, and their power conversion efficiencies (PCEs) have increased from 3.8 to 25.2% within the last decade.^8^, ^9^, ^10^, ^11^, ^12^, ^13^ Typically, metals such as Au,^14^, ^15^ Ag,^16^, ^17^, ^18^ Cu,^19^, ^20^ and Al,^21^, ^22^ are used as contact electrode in PSCs. Noble metal electrodes for high efficiency PSCs have dramatically increased the fabrication cost and seriously hindered their commercial application, because the noble metals are limited as resources and are expensive. Moreover, noble metal electrodes are usually fabricated through rigorous processes using high vacuum and long‐term thermal evaporation in a vacuum chamber. To simplify the fabrication process and reduce the cost of PSCs, carbon‐based materials with inexhaustible properties and excellent stability seem to be preferred choices to replace metal electrodes in PSCs.^23^, ^24^, ^25^, ^26^ However, the photovoltaic performance of PSCs using carbon‐based electrodes remains far behind that of metal electrode‐based devices due to mismatched energy alignment and poor interface contact between the hole transport later (HTL) and electrode.^24^, ^27^, ^28^, ^29^, ^30^

Despite the high efficiency of PSCs using noble metal electrodes, the energy pay‐back period remains quite long due to the poor stability of devices.^31^, ^32^ The single‐time utilization of noble metal electrodes severely wastes limited resources and leads to environmental pollution. A type of monolithic structured PSC using a mesoporous Ni and nanoporous Au contact electrode has been proposed to enable the reuse of contact electrodes.^33^, ^34^ However, these still cannot avoid the complicated and high‐cost fabrication process. They also require a high temperature fabrication^33^ and high‐vacuum thermal evaporation due to the limitations posed by their monolithic device architecture by the post‐treatment required to form the porous structure of a contact electrode.^34^ Moreover, the reported PCEs of ≈10% of those PSCs are very far behind from those of PSCs composed of typical thermal evaporated Au electrodes. PCEs are dramatically decreased after the electrodes are reused and thus the applied electrodes do not significantly contribute to reducing the total energy pay‐back period of the device. Therefore, a new approach is necessary to realize the recyclability of nanoporous metal contact electrode with high specific surface areas. This will enable the use of a facile transferring method of metal contact electrode without sacrificing the merits of solar energy utilization of superior perovskites.

In this study, we successfully developed a new and facile approach to deposit a nanoporous Au film contact electrode using a dry transfer process in PSCs. The high specific surface area of the nanoporous Au electrode results in tight contact with HTL of 2,2,7,7‐tetrakis(N,N‐di‐p‐methoxyphenylamine)‐9,9‐spirobifluorene (spiro‐OMeTAD). This enables a high PCE of 19.0% with a negligible hysteresis of photovoltaic performance in PSCs. The superior properties of the nanoporous Au electrode facilitate recycling, where the same electrode can be used more than 12 times in PSCs. This significantly reduces device fabrication cost and noble Au metal consumption in PSCs. Flexible PSCs (fPSCs) that use nanoporous Au films may be able to sustain a much higher bending durability of ≈98.5% after 1000 bending cycles in comparison with that measured in the usual fPSCs based on the evaporated Au. This is attributed to the nanoporous structure that hinders crack generation and propagation in nanoporous Au films. This study demonstrates the following: the aforementioned novel simplified deposition process, the recycling of the electrode in PSCs using nanoporous Au electrode films, and PSC devices performance.

## 2 Result and Discussion

**Figure 1:**
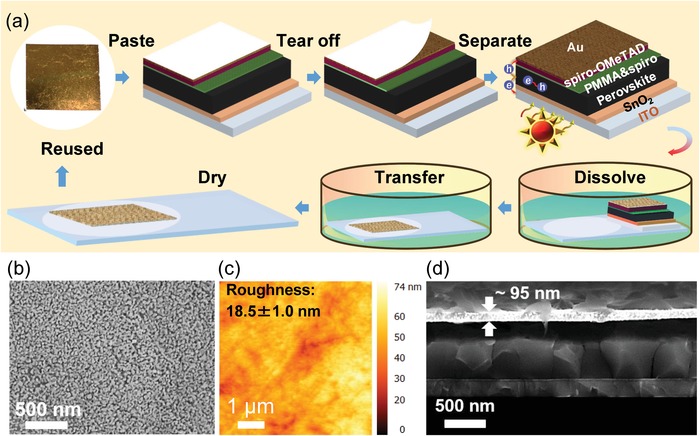
a) Schematic flow of the fabrication and restoration process of the nanoporous Au electrode in PSCs. b,c) Surface morphology and roughness of the nanoporous Au film measured by the SEM and AFM, respectively. d) Cross‐sectional SEM image of a PSC with the nanoporous Au electrode. The scale bar of the SEM and AFM is 500 nm and 1 µm, respectively.

The nanoporous Au has been extensively studied and widely applied in electro‐catalysis or lithium batteries because of its high conductivity, specific surface area, and other merits.^35^, ^36^, ^37^ Therefore, we adopted a nanoporous Au film as an electrode to realize a simple fabrication process and electrode recycling in PSCs based on these superior properties. ≈100 nm of commercial Au_35_Ag_65_ (at%, atomic percentage) thin film was used to fabricate nanoporous Au film by corroding the silver composition using nitric acid, washing it with distilled water and transferring onto membrane film surface. **Figure** **1**a shows the schematic flow of the fabrication and restoring of the nanoporous Au electrode in a PSC. The nanoporous Au on a membrane film was directly attached to the HTL, and the membrane film was then slowly removed using plastic tape to prevent damaging of the nanoporous Au electrode. This process is referred to as fabrication. To restore the electrode after the characterization, the PSC on a glass substrate with a fresh membrane film was placed in acetone in a culture dish to dissolve the perovskite layer and HTL. After these layers were dissolved, the floated nanoporous Au film was transferred to membrane film surface using tweezers. Then, the collected nanoporous Au film on a membrane film was then washed five times with acetone for subsequent utilization. The fabrication and restoring process was repeated as a recycling process. The detailed processes are described in the Experimental section and in videos provided in the Supporting Information.

The architecture of PSC is shown in Figure 1a and consists of a glass substrate/indium tin oxide (ITO)/SnO_2_/Cs_0.05_(MA_0.17_FA_0.83_)_0.95_Pb(I_0.83_Br_0.17_)_3_‐perovskite/poly(methyl methacrylate) (PMMA):spiro‐OMeTAD mixed surface passivation/spiro‐OMeTAD/Au electrode (evaporated and nanoporous Au film). The thin spacer of a PMMA:spiro‐OMeTAD layer was inserted between the perovskite and HTL (see Figures S1–S4 and Tables S1 and S2, and Note S1 in the Supporting Information). Interestingly, the perovskite layer that was passivated with PMMA:spiro‐OMeTAD could not be dissolved in ethanol solvent despite being kept it for 1 h, indicating that the PMMA:spiro‐OMeTAD thin film may prevent the perovskite from dissolving during the nanoporous Au film transferring process, as shown in Figure S5 (Supporting Information). The PCE of the evaporated Au PSCs (evap‐Au/PSCs) was enhanced by as much as 20.4% with a negligible hysteresis after the PMMA:spiro‐OMeTAD layer was implemented with an optimized concentration ratio of 10:1.5 mg mL^−1^ (wt:wt).

Figure 1b shows the surface morphology of the nanoporous Au film as characterized by scanning electron microscopy (SEM). The nanoporous Au film consists of co‐adjacent nanoporous structures with a narrow pore‐size distribution, which is in contrast with the nanocrystal structure and large distribution in evaporated Au film, as shown in Figure S6a (Supporting Information). The elemental composition of nanoporous Au film was also characterized by energy‐dispersive X‐ray (EDX) spectroscopy, as shown in Figure S7a,b (Supporting Information). The main composition of the nanoporous Au film is Au and Ag at an atomic ratio of 3.3:1. Au is uniformly distributed according to the EDX mapping shown in Figure S7b (Supporting Information), which severely impacts the electrical conductivity of the nanoporous Au film.

Figure 1c and Figure S6b (Supporting Information) present surface roughness evaluations of the nanoporous and evaporated Au films, respectively, measured using atomic force microscopy (AFM). The nanoporous Au film with an average surface roughness of 18.5 ± 1.0 nm was slightly larger than that of the evaporated Au film (8.8 ± 0.9 nm). The slightly larger surface roughness of nanoporous Au film does not affect the tight interface contact between spiro‐OMeTAD and the Au electrode film. The contact properties of Au film with spiro‐OMeTAD as HTL were evaluated by examining its cross‐sectional SEM image, as shown in Figure 1d and Figure S6c (Supporting Information). Note that the nanoporous Au film was in tight contact with the HTL after a simple dry transfer process was conducted and without requiring vacuum thermal deposition. The film was also much improved over that derived from carbon‐based electrode stacking.^27^, ^28^, ^38^, ^39^ The thickness of the nanoporous Au was ≈95 nm, as estimated from the cross‐sectional SEM image. This thickness was comparable to that of the evaporated Au film (≈80 nm).

The evaluated conductivity and specific surface area of both Au films are shown in **Table** **1**. The sheet resistance of the nanoporous Au film was sufficiently low (11.3 ± 0.5 Ω) as compared to that of the evaporated Au film (5.3 ± 0.5 Ω), indicating that PSCs that use nanoporous Au films can achieve a high short‐circuit current density (*J*_SC_) without requiring vacuum thermal deposition. The value of the specific surface area of the nanoporous Au film was very high (i.e., 133.0 m^2^ g^−1^) as compared to that of evaporated Au film (1.7 m^2^ g^−1^), which is critical for the transfer and contact between HTL and the Au electrode, as shown in Figure S8 (Supporting Information). Thus, a high photovoltaic performance can be achieved in nano‐Au/PSCs using a simple transfer process. In addition, high conductivity and an extremely high specific surface area enable the nanoporous Au electrode in PSCs to be recycled, a process which is described later.

**Table 1:** Sheet resistance and specific surface area of evaporated and nanoporous Au films

Sample	Sheet resistance [Ω]	Specific surface area [m^2^ g^−1^]
Evaporated Au film	5.3 ± 0.5	1.7
Nanoporous Au film	11.3 ± 0.5	133.0

**Figure 2:**
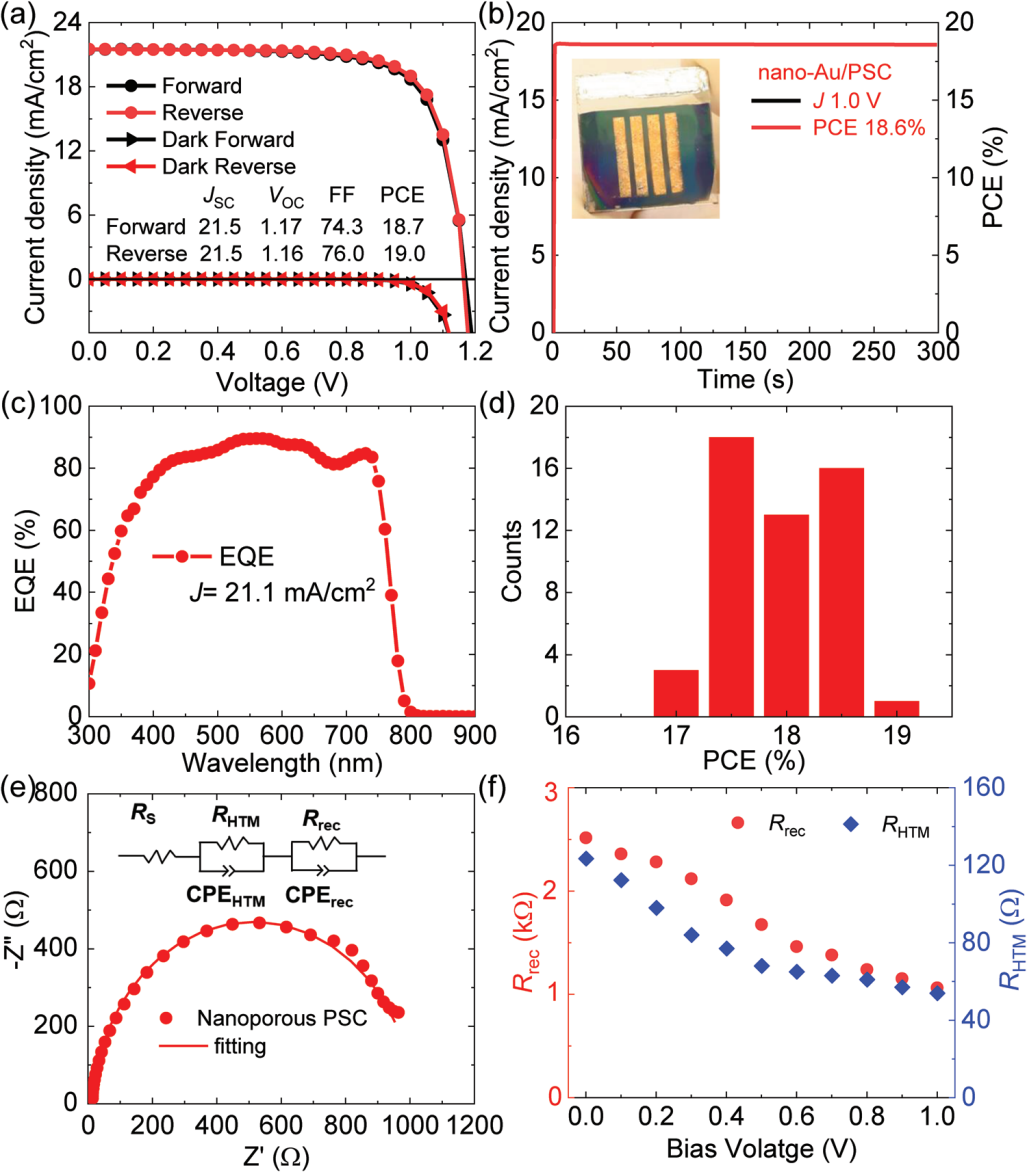
a) *J*–*V* curves of nano‐Au/PSCs measured at 50 mV s^−1^. The black and red circles refer to data derived from forward and reverse scans, respectively. The masked active area of each cell is ≈0.03 cm^2^. b,c) SPO and EQE of nano‐Au/PSCs. The photograph of nano‐Au/PSCs was inserted in the SPO. d) Statistical distribution of the PCEs of 50 nano‐Au/PSCs. e) Nyquist plots of the nano‐Au/PSCs under one sunlight illumination condition. The equivalent circuit model for the simulated curve is shown in the inset. f) *R*_rec_ and *R*_HTM_ as a function of bias voltage in the nano‐Au/PSCs.

**Figure** **2**a shows the current density‐voltage (*J*‒*V*) curves of our best performance PSC with the nanoporous Au electrode (nano‐Au/PSCs). The nano‐Au/PSCs exhibited a high PCE of 18.7% (19.0%) with a high open‐circuit voltage (*V*_OC_) of 1.17 V (1.16 V), a *J*sc of 21.5 mA cm^−2^ (21.5 mA cm^−2^), and a fill factor (FF) of 74.3% (76.0%), as measured at forward (reverse) scanning. These results were nearly comparable to the highest efficiency carbon‐based PSCs fabricated by complicated and multistep preparation processes^27^, ^38^, ^39^, ^40^, ^41^, ^42^ and were much better than those of the monolithic structure of porous Ni and Au electrodes in PSCs produced using a complicated fabrication procedure.^33^, ^34^ We evaluated the stabilized power output (SPO) of nano‐Au/PSCs with a bias voltage of 1.0 V under the maximum power point (MPP), as shown in Figure 2b. A photograph of nano‐Au/PSCs has also been inserted in the figure. The nano‐Au/PSC achieved a high SPO of 18.6% and sustained a stable value during evaluation. The external quantum efficiency (EQE) of nano‐Au/PSCs was characterized using incident‐photon‐to‐current‐efficiency spectroscopy. Figure 2c shows the EQE spectrum of nano‐Au/PSCs exhibited an average value of 85.1% in the range of 400–740 nm with an integrated current density of 21.1 mA cm^−2^. We statistically evaluated the reproducibility of 50 nano‐Au/PSCs devices under reverse scanning, as shown in Figure 2d and Figure S9 (Supporting Information). The nano‐Au/PSCs exhibited a narrow distribution of all the photovoltaic parameters. Noted that the photovoltaic performance and the reproducibility of the nano‐Au/PSCs were much higher and narrower than those of the monolithic structure of nanoporous Au PSCs,^34^ the fact that the thermal evaporating (long and high‐vacuum) condition was not used to deposit the Au electrode. Interestingly, the nano‐Au/PSCs maintained nearly the same storage stability as the evap‐Au/PSCs under ambient conditions with a humidity of ≈25%, indicating that the storage stability of nano‐Au/PSCs was not obviously affected without the thermal evaporation process and anhydrous ethanol being used during nanoporous Au transfer, as shown in Figure S10 (Supporting Information).

Impedance spectroscopy was used to evaluate the carrier transport properties in PSCs with the nanoporous and evaporated Au electrodes. Figure 2e and Figure S4e (Supporting Information) show the Nyquist plots of the nano‐ and evap‐Au/PSCs. The impedance spectra consisted of two features in the high‐ and low‐frequency regions derived from the contributions of carrier transport from perovskite to the HTL and the carrier recombination process,^11^, ^43^, ^44^, ^45^ respectively. The equivalent circuit to reproduce the impedance spectra is shown in the insets of Figure 2e and Figure S4e (Supporting Information). Figure 2e and Figure S4e (Supporting Information) show the simulated curves of impedance spectra of two types of PSCs. The simulated curves effectively reproduce the experimental results of impedance spectra in both PSCs, and the parameters of hole transport resistance (*R*_HTM_) and recombination resistance (*R*_rec_) were evaluated based on this analysis.^45^

The evaluated *R*_rec_ and *R*_HTM_ as a function of bias voltage under sunlight illumination condition are shown in Figure 2f. *R*_rec_ and *R*_HTM_ of nano‐Au/PSCs continuous decrease as the bias voltage increased, which was similar to observations in previous studies.^11^, ^46^, ^47^ The continuous reduction of *R*_rec_ and *R*_HTM_ indicates that the possibility of carrier recombination became severe and the carrier extraction ability decreases with increasing bias voltages.^11^, ^46^, ^47^ The *R*_rec_ and *R*_HTM_ of nano‐Au/PSCs show slightly lower and higher values, respectively, as compared to those in the evap‐Au/PSCs, as shown in Figure S4d–f (Supporting Information). This was because of the relatively weak contact between the HTL and nanoporous Au film.

The environmental stability of PSCs in the moisture and oxygen is insufficient for industrial application, which is a critical and ongoing issue in PSC studies. The Au electrode is usually used only once and frequently discarded after degradation of perovskite photoactive layers in PSCs. The nanoporous Au film can be directly applied to the recycling part of PSCs without any additional processing, which is another advantage in addition to the simple and facile fabrication by dry transfer. We studied the recycling of the nanoporous Au electrodes in PSCs to reduce fabrication cost, noble Au metal resource waste and environmental pollution.

**Figure 3:**
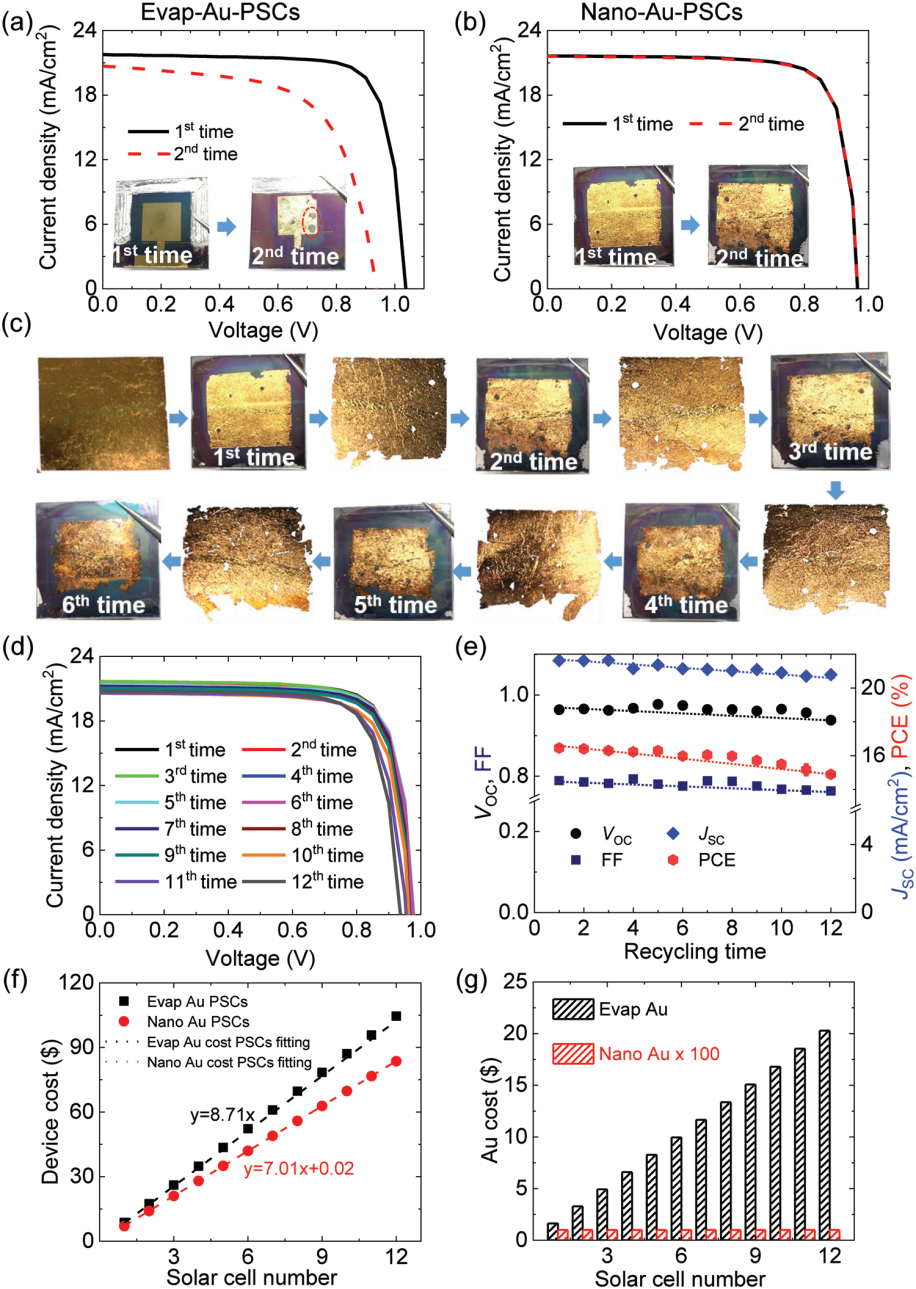
a,b) *J*–*V* curves of evap‐ and nano‐Au/PSCs; the evaporated and nanoporous Au electrodes are reused (two recycling process, where the red dotted lines refer to recycled photovoltaic performance). The photographs of the first‐ and second‐time used evaporated and nanoporous Au films in PSCs were inserted in the *J*–*V* curves. c) Photographs of nanoporous Au film recycling in the PSCs with 6 times. The size of the PSC substrate is 25 × 25 mm^2^. d) *J*–*V* curves of nano‐Au/PSCs, where the nanoporous Au electrode was reused multiple times (12 times recycling). e) Changes in photovoltaic parameters in the nano‐Au/PSCs during 12 times recycling. f) Calculated fabrication cost of evap‐ and nano‐Au‐PSCs as a function of the recycled utilization of electrodes. *X* and *Y* in the figure denote the number of PSCs devices, and calculated cost of PSCs, respectively. g) Noble Au consumption of evaporated and nanoporous Au films as the number of iterations of recycled utilization increased.

**Figure** **3**a shows *J*‒*V* curves and photographs of the evap‐Au/PSCs during recycling of the evaporated Au film. The PCE of the evap‐Au/PSCs prior to the recycling (1st time) was 17.7%, as shown in Table S3 (Supporting Information). The photovoltaic performance and its PCE drastically decreased in the evap‐Au/PSCs after recycling (2nd time) because of the major reduction of FF and *V*_OC_, as shown in Figure 3a. The major decrease in photovoltaic performance after recycling of the evaporated Au electrode was caused by folds and tears in the vulnerable film when the evaporated Au electrode was transferred to fresh PSCs, as shown in Figure S11a (Supporting Information).

Figure 3b shows *J*–*V* curves and photographs of the nano‐Au/PSCs when a recycled nanoporous Au electrode was used. The PCE of the nano‐Au/PSCs prior to recycling (1st time) was 16.5%. The photovoltaic performance and its PCE (16.5%) are maintained and did not obviously decrease in the nano‐Au/PSCs even after the electrode is recycled (2nd time), as shown in Table S3 (Supporting Information). Noted that the nano‐Au/PSC sustained a high photovoltaic performance after the nanoporous Au electrode was recycled, which is much better than that of the recycled evap‐Au/PSCs, monolithic structure device^33^, ^34^ and the recycled glass‐FTO/TiO_2_‐electron transport‐layer PSCs that underwent high‐temperature postannealing prior to reuse.^48^

We conducted further processes to realize continuous recycling of the nanoporous Au electrodes in PSCs, as shown in Figure 1a and described in the Supporting Information. Figure 3c shows photographs of nanoporous Au that was recycled 6 times. Considerably higher recycled nanoporous Au is shown in Figure S11b (Supporting Information), where the active area of nanoporous Au remained obviously unchanged after 6 times recycling. Figure 3d shows *J*–*V* curves of the nano‐Au/PSCs measured under reverse scanning during 12 times recycling. The nano‐Au/PSC maintained a high photovoltaic performance and exhibited only slight changes in the PCE after 12 times recycling. The changes in the photovoltaic parameters of the nano‐Au/PSC as a function of recycling times are shown in Figure 3d. The slow decline in the photovoltaic performance of the nano‐Au/PSC derives from the main deterioration of the FF and *J*_SC_ and slight decrease in *V*_OC_, as shown in Figure 3d. To realize continuous recycling, a flat surface must be retained, and the electrode is then transferred to the subsequent PSCs without any folds; this is confirmed by the cross‐sectional SEM images in Figure S12 (Supporting Information). A SEM image of the nano‐Au/PSCs reveals the tight contact between the Au electrode and HTL without any gaps or holes, which contrasts with that of the evap‐Au/PSCs, in which the evaporated Au electrode has folds. The calculated fabrication cost of PSCs and the amount of material consumption of Au are shown in Figure 3f,g, and in Table S4 (Supporting Information). Figure 3f shows a comparison of the calculated fabrication cost of PSCs using recycled nanoporous Au and evaporated Au films. The increment cost of evap‐Au/PSCs as compared to nano‐Au/PSCs increases as the recycled times increase, indicating the merits of nanoporous Au recycling in term of considerable savings. In addition, the total cost of evaporated Au film is more than 1000 times that of nanoporous Au film after 12 times recycling as electrodes, as shown in Figure 3g. Therefore, the recycled nanoporous Au film decreased the fabrication cost of PSCs, which also contributes to reducing noble Au metal consumption and preventing environmental pollution.

We considered the physical reasons for the slight reduction in the photovoltaic performance of nano‐Au/PSCs during recycling. Figure S10b (Supporting Information) shows a series of photographs of the nanoporous Au film during each transfer process. The edges of the nanoporous Au film gradually broke, and the area of the film corresponding to the active area decreased with each recycling instance. The elemental spectrum of the 12 times reused nanoporous Au film as analyzed by SEM‐EDX is shown in Figure S7c,d (Supporting Information). The main elements of the reused nanoporous Au film are Au and Ag, and their atomic ratio is 3.9:1, which is similar to that of the fresh film, as shown in Figure S7c (Supporting Information). In addition, a small amount of iodide (I) was also detected, where the atomic ratio of Au, Ag, and I is 44.4:11.3:1. I is derived from the dissolving process of the perovskite layer and is uniformly distributed on the reused nanoporous Au film according to the EDX maps in Figure S7d (Supporting Information). Residual I is likely another cause of the slight decline in photovoltaic performance of the nano‐Au/PSCs, as the decrease in *J*_SC_ and FF derive from the reduced conductivity of the nanoporous Au film after several recycling processes. The sheet resistance of the nanoporous Au film was slightly increased to as much as 14.5 ± 1.2 Ω as compared with that of the fresh film, which derives from residual chemicals such as I, as confirmed by EDX characterization in Figure S8 (Supporting Information). However, the nanoporous Au film still sustains sufficiently high conductivity even after 12 times utilization for high photovoltaic performance of PSCs.

**Figure 4:**
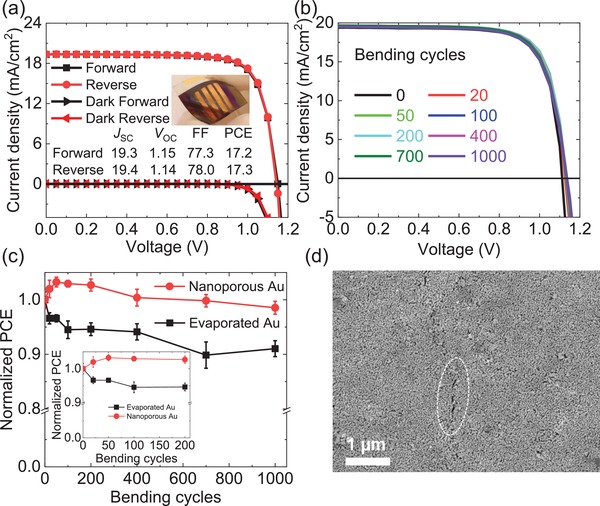
a) *J*–*V* curves of fPSCs with a nanoporous Au electrode. The active area of each device is ≈0.03 cm^2^. b) *J*–*V* curves of fPSCs under various bending cycles at a bending radius of 5 mm in reverse scanning. c) Normalized PCE in nano‐ and evap‐Au/fPSCs as a function of bending cycles. d) Surface morphology of the nanoporous Au electrode after 400 bending cycles at a bending radius of 5 mm. The scale bar for the SEM image is 1 µm.

We also introduced the nanoporous Au film in flexible PSCs (nano‐Au/fPSCs) to enhance the bending durability of the flexible devices. **Figure** **4**a shows *J*–*V* curves of the best fPSCs using the nanoporous Au film electrodes. The *J*–*V* curves show a negligible hysteresis under forward and reserve scanning. The nano‐Au/fPSCs achieved a high PCE of 17.2% (17.3%) under forward (reverse) scanning, which was slightly lower that the PCE of 18.2% (18.3%) in evaporated Au fPSCs (evap‐Au/fPSC), as shown in Figure S13a (Supporting Information). The nano‐Au/fPSCs exhibited a stable value of SPO of 17.2% under the MPP with continuous light irradiation, as shown in Figure S13b (Supporting Information), which was much higher than that of the carbon based electrode fPSCs.^28^, ^29^, ^40^, ^49^, ^50^, ^51^ The reproducibility of the nano‐Au/PSCs and evap‐Au/fPSCs was evaluated using 20 and 10 fabricated devices, respectively, and the statistical distribution of the photovoltaic parameters is summarized in Table S4 (Supporting Information).

Harsh bending tests were conducted on fPSCs, and the process is shown in a video provided in the Supporting Information. The *J*–*V* curves of the nano‐Au/fPSCs as a function of bending cycles at a bending radius of 5 mm are shown in Figure 4b; the curves underwent practically no changes up to 1000 bending cycles, thus demonstrating the superior bending durability of the nano‐Au/fPSCs. By contrast, the *J*–*V* curves of the evap‐Au/fPSC substantially changed due to a slow decrease in FF and *J*_SC_, as shown in Figure S13d,f (Supporting Information), respectively. Figure 4c shows the averaged bending durability of normalized PCEs as a function of bending cycles in four devices (nano‐ and evap‐Au/fPSCs). The nano‐Au/PSCs sustained a high average PCEs of ≈98.5% as compared with that of ≈91.0% in evap‐Au/fPSCs after 1000 bending cycles, which is comparable to the carbon electrode‐based fPSCs.^28^, ^40^, ^49^, ^50^, ^51^, ^52^ The photovoltaic parameters (*J*_SC_, *V*_OC_, and FF) of the nano‐ and evap‐Au/fPSCs are shown in Figure S13d–f (Supporting Information).

Interestingly, the normalized PCE in the nano‐Au/fPSCs slightly increased for bending cycles of <50 times and slightly decreased after 100 cycles, as shown in the inset of Figure 4c. The increase in the normalized PCE may have derived from the enhanced contact between the HTL and nanoporous Au electrode during bending cycles, as the relatively large roughness of the surface of the nanoporous Au film produced some nanoscale void spaces between the HTL just after the transfer, and the embedded nanoscale void spaces are released during initial bending cycles (<50 times). As the number of bending cycles increases, cracks in the Au electrode were generated in both the nano‐Au/PSCs and evap‐Au/fPSCs. However, we found that the surface of the nanoporous Au electrode remained unchanged like that of the fresh electrode, and tiny cracks were much shorter than those of the evaporated Au electrode (see the SEM images in Figure 4d; Figure S14, Supporting Information). The porous structure largely released the bending stress to the co‐adjacent structure and prevented crack propagation, thus enabling the electrode to retain high carrier transport properties during bending. Therefore, the nano‐Au/fPSCs had sustained higher photovoltaic performance and superior bending durability, which is another promising advantage of the nanoporous Au electrode.

## 3 Conclusion

We successfully introduced a nanoporous Au film as a contact electrode in rigid and flexible planar PSCs to replace contact metal electrodes deposited by thermal evaporation, which is a complicated, rigorous, and expensive method. By using a nanoporous Au electrode, we were able to recycle the Au electrode in the nano‐Au/PSCs more than 12 times, reducing the fabrication cost of PSCs and noble Au metal consumption and achieving a high PCE of 19.0% and only a slight degradation of photovoltaic performance during recycling. In addition, we demonstrated a high PCE of 17.3% in nano‐Au/fPSCs exhibiting superior bending durability (≈98.5%) after 1000 bending cycles at a bending radius of 5 mm. Applying the nanoporous Au electrode introduces a facile, feasible and effective approach for replacing the complicated and rigorous metal electrode deposition process that is usually necessary to create PSCs with high photovoltaic performance. It also contributes to the acceleration of the industrial application of PSCs. The continuous recycling of noble metal electrodes in photovoltaic devices significantly reduces the device fabrication cost and minimizes resources waste and environmental pollution, thus impacting a wide range of research fields and revolutionizing the photovoltaic industry.

## Conflict of Interest

The authors declare no conflict of interest.

## Supporting information

Supporting Information

Supplemental Video 1

Supplemental Video 2
