# Supplementary material for: Recycled Utilization of a Nanoporous Au Electrode for Reduced Fabrication Cost of Perovskite Solar Cells
Source: Adv Sci (Weinh). 2020 Jan 30;7(6):1902474. doi: 10.1002/advs.201902474 (PMC7080531; doi:10.1002/advs.201902474)
Supplement: Supplementary file 1 — Supporting Information [file ADVS-7-1902474-s001.pdf]

Copyright WILEY-VCH Verlag GmbH & Co. KGaA, 69469 Weinheim, Germany, 2020.

ADVANCED SCIENCE
Open Access

Supporting Information

for *Adv. Sci.,* DOI: 10.1002/advs.201902474

Recycled Utilization of a Nanoporous Au Electrode for Reduced Fabrication Cost of Perovskite Solar Cells

Fengjiu Yang, Jinzhe Liu, Zheng Lu, Pengfei Dai, Tomoya Nakamura, Shenghao Wang, Luyang Chen, Atsushi Wakamiya,* and Kazunari Matsuda**

Copyright WILEY-VCH Verlag GmbH & Co. KGaA, 69469 Weinheim, Germany, 2018.

Supporting Information

Recycled Utilization of a Nanoporous Au Electrode for Fabrication Reduced Cost of Perovskite Solar Cells

Fengjiu Yang, Jinzhe Liu, Zheng Lu, Pengfei Dai, Tomoya Nakamura, Shenghao Wang, Luyang Chen, Atsushi Wakamiya*, and Kazunari Matsuda**

Copyright WILEY-VCH Verlag GmbH & Co. KGaA, 69469 Weinheim, Germany, 2016.

Supporting Information

Recycled Utilization of a Nanoporous Au Electrode for Reduced Fabrication Cost of Perovskite Solar Cells

Fengjiu Yang, Jinzhe Liu, Zheng Lu, Pengfei Dai, Tomoya Nakamura, Shenghao Wang, Luyang Chen, Atsushi Wakamiya*, and Kazunari Matsuda**

Experimental section

Materials. All chemicals and solvents were directly used as received without further refinement. The indium tin oxide (ITO) substrates (10 Ω) were purchased from GEOMATEC Co., Ltd. SnO_2 nanoparticle (15 wt.%) in H_2O colloidal dispersion with a particle size of 10–15 nm was purchased from Alfa Aesar. Lead (II) iodide (PbI_2, 99.99%), lead (II) bromide ($PbBr_2$, 99%), methylammonium bromide (MABr, >98.0%) formamidinium iodide (FAI, >98.0%), and cesium iodide (CsI, 99.9%) were all purchased from Tokyo Chemical Industry Co., Ltd. Poly(methyl methacrylate) (PMMA) (M_w≈15000, powder), 4-tert-butylpyridine (tBP), and Co(4-tertbutylpyridyl-2-1H-pyrazole)3·3-bis(trifluoromethanesulfonyl)imide (TFSI) were purchased from Sigma-Aldrich Co., Ltd. The solvents of *N*,*N*-dimethylformamide (DMF), dimethyl sulfoxide (DMSO), chlorobenzene, toluene, ethanol and isopropanol (IPA) were dried prior to use. 2,2′,7,7′-Tetrakis(*N*,*N*-di-*p*-methoxyphenylamino)-9,9′-spirobifluorene (spiro-OMeTAD) was ordered from Merck Co., Ltd. Lithium-TFSI (Li-TFSI) salt powder, acetone, and other solvents were purchased from

Wako Chemical Co., Ltd. and used as received without treatment unless otherwise noted. The membrane films were ordered from Merck Millipore Ltd.

ITO substrate preparation. The ITO substrates were treated using the same methods as in our previous study.[1] The SnO_2 electron transport layer was deposited through the same approach as previously reported using SnO_2 nanoparticles with a 5-wt.% concentration.[1] The prepared ITO/SnO_2 substrates were stored in a dry cabinet for the fabrication of perovskite.

Nanoporous Au film preparation. A commercial $Au_{35}Ag_{65}$ (at.%, atomic percentage) thin film with ca. 100-nm thickness was used to fabricate a nanoporous Au film using a dealloying approach with a concentration of 69-wt.% of HNO_3 solution for 6 h under ambient conditions, which formed the nanoporous structure after the composition of silver was corroded due to the weak resistance of nitric acid.[2–5] The residual chemical substances within the pore channels or on the surface of the nanoporous film were rinsed away using deionized water three times. The obtained nanoporous Au films were transferred to the membrane surface, dried under ambient conditions without any treatments, and stored in a cleaning box for subsequent transference onto perovskite solar cells (PSCs).

PSCs fabrication. All PSC fabrications were completed in a nitrogen glovebox. The perovskite film was prepared using the same fabrication approach as in our previous report.[1,6] The perovskite surface was passivated using PMMA (10 mg/mL).[7] The mixed PMMA and spiro-OMeTAD passivation was conducted by dissolving spiro-OMeTAD and PMMA in chlorobenzene separately. The PMMA:spiro-OMeTAD solutions with various concentration ratios (10:(0, 0.5, 1.0, 1.5, 2.0, 3.0 and 5.0) mg/mL) were prepared by mixing the PMMA and spiro-OMeTAD solutions. Then, the PMMA:spiro-OMeTAD solution was deposited using the same approach as that for PMMA. The hole transport layer (HTL) was

deposited using the same method as explained in our previous study.[7] Finally, the ca. 80 nm Au electrode was deposited by thermal deposition in vacuum.

Nanoporous Au film transfer. All transfer processes were performed under ambient conditions. The membrane-film-suspended nanoporous Au was slowly attached to the HTL surface, where 200 μL of anhydrous ethanol was deposited on back-side of the membrane film to wet the nanoporous Au film and HTL. The ethanol was then discarded by spin coating the sample at 5000 rpm for 60 s. The membrane film was slowly removed using plastic tape to prevent damaging the nanoporous Au film. The PSCs were kept in vacuum to enhance the contact between the nanoporous Au film and HTL, and the residual ethanol was removed. To check the recycling capabilities of the nanoporous Au film, the HTL and perovskite were dissolved using acetone, and the nanoporous Au film was transferred to a fresh membrane film surface in a culture dish. The transferred nanoporous Au film was washed by acetone five times to reduce the any remaining residual chemicals. The detailed transfer process is provided in a video and shown in Figure 1.

Characterization. All characterization and evaluation procedures were conducted under ambient condition unless other noted.[7] The sheet resistance of evaporated and nanoporous Au films were characterized by a four-point-probe (SR4-S, Astellatech Co.). Photoluminescence (PL) microscopy was performed using a 532-nm semiconductor laser with an incident power of 110 nW in a micro-Raman setup (Ramantouch, Nanophoton Co.). The time-resolved PL was characterized using a single-photon counting technique with a 520-nm semiconductor laser having an incident power of ca. 10 nW. Energy dispersive X-ray spectroscopy (EDX) was conducted using a field-emission scanning electron microscope (SEM, S-4800 Hitachi High-Technologies Co.) instrument. The specific surface area was measured using nitrogen adsorption–desorption isotherm (Autosorb iQ3, Quantachrome

instrument). Impedance spectroscopy was conducted under sunlight condition with various bias voltages using the platform of an electrochemical analyzer (ALS/HCH, 660 EAW).

Supplementary note

The PMMA layer was introduced to passivate the perovskite film surface.[7] The PMMA passivated perovskite solar cells (PMMA/PSCs) exhibited a much higher photovoltaic performance than that of standard PSCs without a PMMA passivation layer, as shown in Table S1. However, the hysteresis of the current density–voltage (*J–V*) curves in the PMMA/PSCs still remained, which was similar to our earlier observations.[7] We attempted to mix a low concentration of 2,2,7,7-tetrakis(N,N-di-p-methoxyphenylamine)-9,9-spirobifluorene (spiro-OMeTAD) into the PMMA to reduce the carrier accumulation at interface of the PMMA under reverse scanning.

Optical characterizations, specifically absorption, photoluminescence, and time-resolved PL, of perovskites were conducted. The PL spectra and decay clearly showed that the PMMA:spiro-OMeTAD passivated perovskite worked much more effectively than the perovskite film without passivation, as shown in Figure S1. The surface and cross-sectional morphology and surface roughness were also evaluated, as shown in Figures S2 and 3. Figure S4a–c show the photovoltaic performance of PSCs with PMMA and PMMA:spiro-OMeTAD passivation layers. The electrochemical properties of the PSCs with PMMA and PMMA:spiro-OMeTAD are also shown in Figure S4d–f. We optimized the concentration of spiro-OMeTAD in the PMMA and found that the photovoltaic performance of PSCs with a PMMA:spiro-OMeTAD passivation layer was maximized at a concentration of 10:1.5 mg/mL of PMMA and spiro-OMeTAD, as shown in Table S1. Thus, this ratio of PMMA and spiro-OMeTAD was employed in the PSCs with the nanoporous Au electrode.

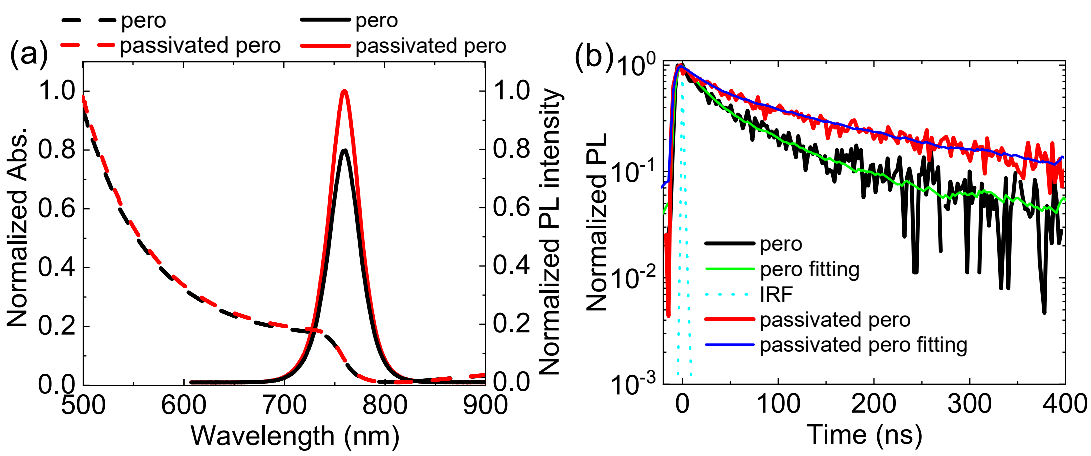

Figure S1. (a) Optical absorption and PL spectra of perovskite film without and with a PMMA:spiro-OMeTAD passivation layer (10:1.5 mg/mL). (b) Time-resolved PL of the perovskite film without and with the PMMA:spiro-OMeTAD passivation layer.

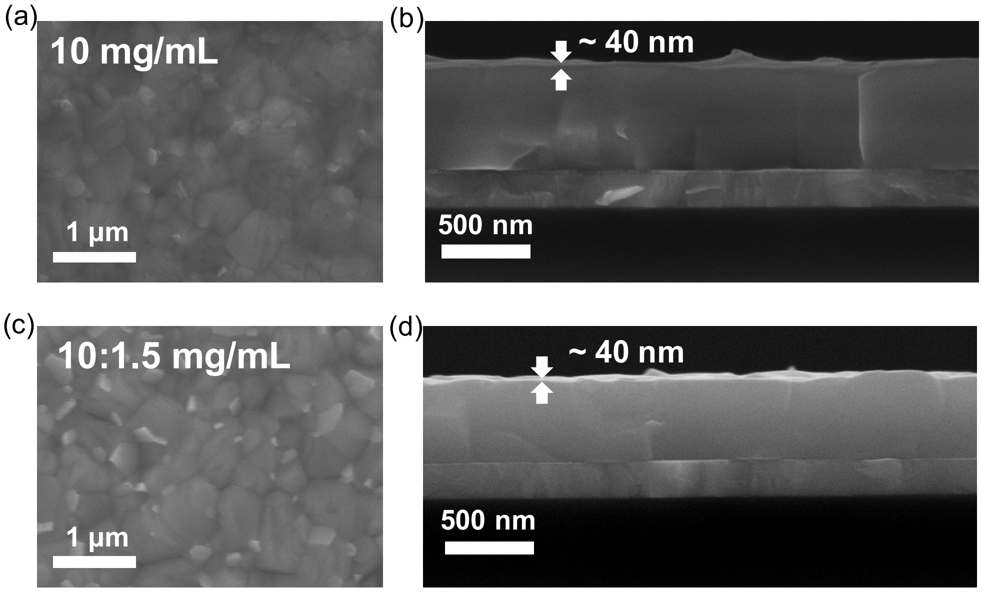

Figure S2. Surface and cross-sectional SEM images of perovskites passivated by PMMA and PMMA:spiro-OMeTAD passivation layers. (a, b) Perovskite passivated by PMMA (10 mg/mL), and (c, d) perovskites passivated by PMMA:spiro-OMeTAD (10:1.5 mg/mL), respectively. The scale bar is 1 μm and 500 nm in the surface and cross-sectional images, respectively.

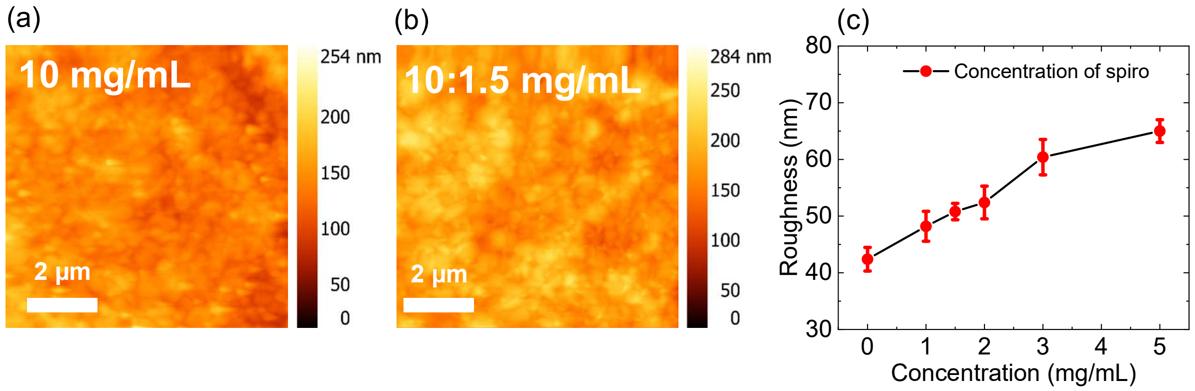

Figure S3. Surface morphology of perovskites passivated by PMMA and PMMA:spiro-OMeTAD layers. (a, b) AFM images of perovskites passivated by PMMA (10 mg/mL) and PMMA:spiro-OMeTAD (10:1.5 mg/mL), respectively. The scale bar is 2 µm. (c) Surface roughness as a function of the ratio of PMMA:spiro-OMeTAD.

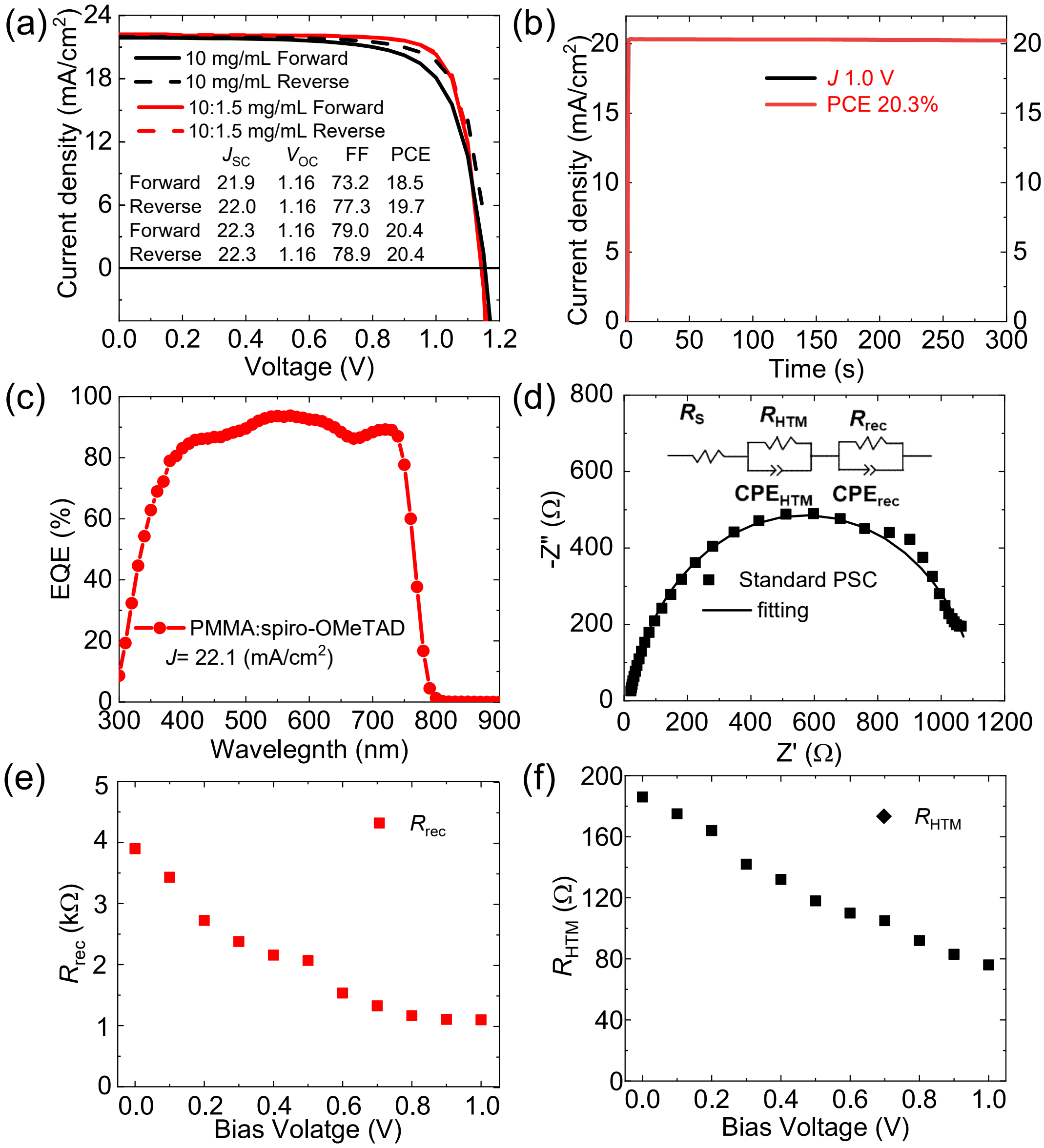

Figure S4. (a) *J–V* curves of evap-Au/PSCs with PMMA and PMMA:spiro-OMeTAD passivation layers. (b) SPO of PSCs with a PMMA:spiro-OMeTAD layer. A photograph of the PSC is also shown in the inset. (c) EQE of PSCs with a PMMA:spiro-OMeTAD layer. (d) Nyquist plots of evap-Au/PSCs at a bias voltage of 1.0 V and sunlight illumination. The simulated equivalent circuit is shown in the inset. (e, f) R_{rec} and R_{HTM} as a function of bias voltage in evap-Au/PSCs.

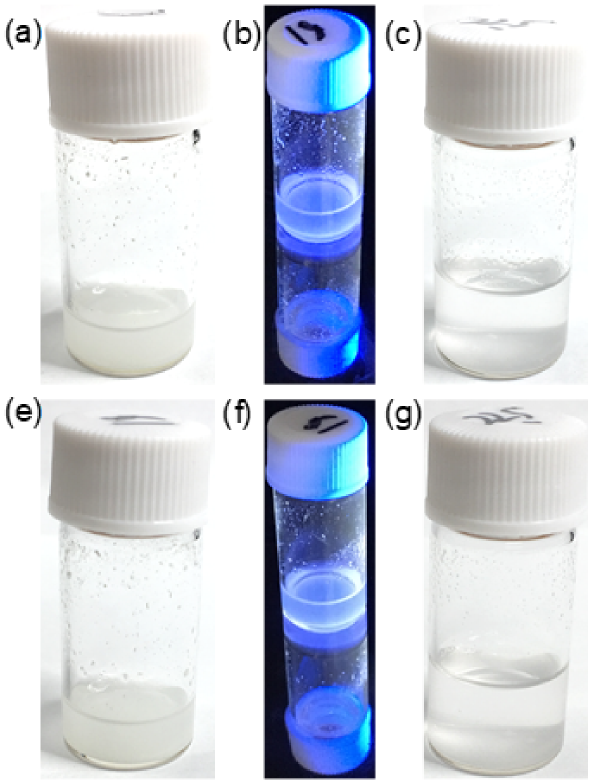

Figure S5. Photographs of PMMA and spiro-OMeTAD dissolved in ethanol solution. (a–c) Fresh chemicals of PMMA (20 mg/mL) and spiro-OMeTAD (10 mg/mL) in ethanol solution. (e–g) PMMA and spiro-OMeTAD in ethanol solution after one hour. (b) and (f) solutions of spiro-OMeTAD were soaked in UV light at 245-nm wavelength. The diameter of the bottle was 10 mm. The PMMA and spiro-OMeTAD did not dissolve in the ethanol solvent even after one hour, indicating that the PMMA and spiro-OMeTAD thin film can prevent the perovskite layer from dissolving during the nanoporous Au film transfer process.

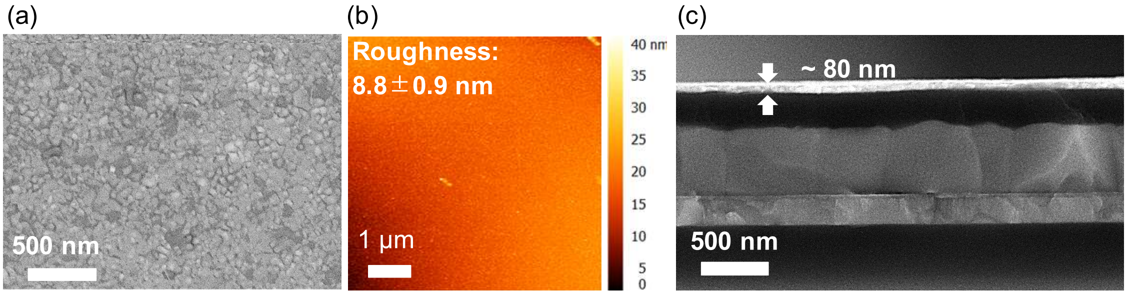

Figure S6. (a) SEM image of the surface morphology of the evaporated Au film. (b) Surface roughness of the evaporated Au film as measured by the AFM. (c) Cross-sectional SEM image of evap-Au/PSCs. The scale bar of the SEM and AFM is 500 nm and 1 µm, respectively.

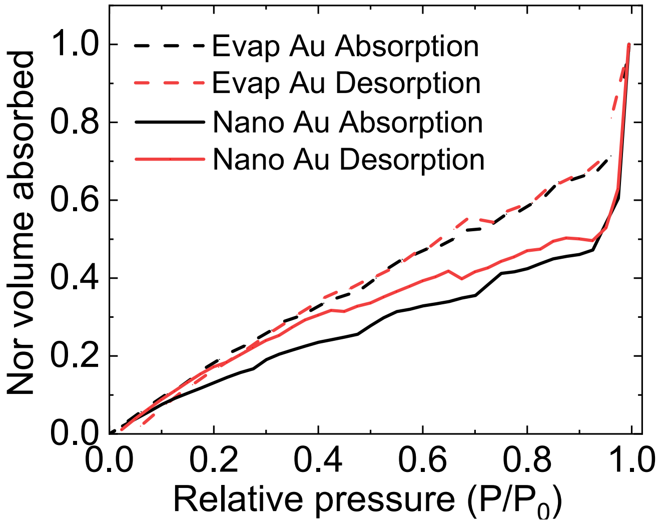

Figure S7. Nitrogen adsorption–desorption isotherms of the evaporated and nanoporous Au films, respectively.

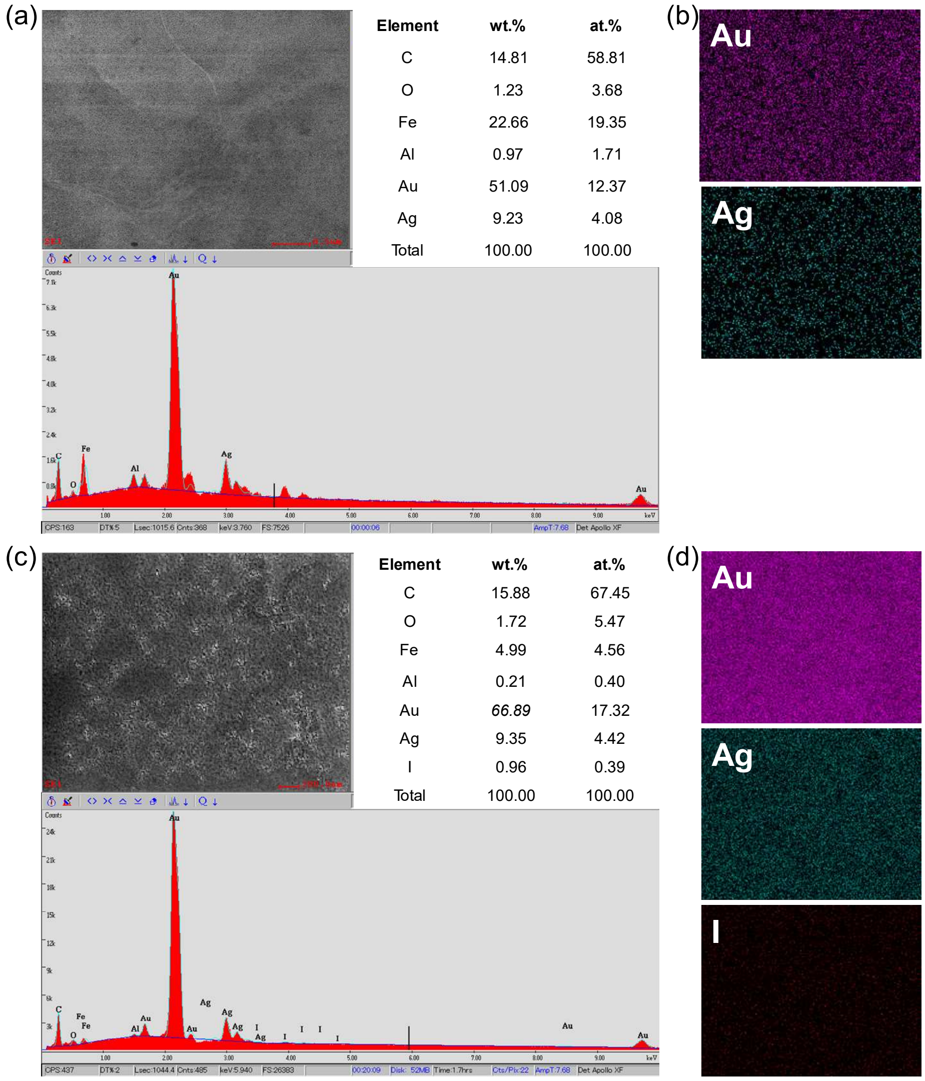

(a)

Element	wt.%	at.%
C	14.81	58.81
O	1.23	3.68
Fe	22.66	19.35
Al	0.97	1.71
Au	51.09	12.37
Ag	9.23	4.08
Total	100.00	100.00

(c)

Element	wt.%	at.%
C	15.88	67.45
O	1.72	5.47
Fe	4.99	4.56
Al	0.21	0.40
Au	66.89	17.32
Ag	9.35	4.42
I	0.96	0.39
Total	100.00	100.00

Figure S8. EDX spectra of fresh and recycled (12 times) nanoporous Au films. (a) Atomic percentages of the main elements (C, O, Fe, Au and Ag) in the nanoporous Au film are summarized in the inserted table. The evaluated atomic ratio of Au and Ag is 3.3:1. The scale bar is 500 nm. (b) EDX mapping of Au and Ag. A uniform distribution of Au and Ag is observed. (c) Atomic percentages of the main elements (C, O, Fe, Au and Ag) of the nanoporous Au film after recycling 12 times are summarized in the inserted table. The evaluated atomic ratio of Au, Ag and I is 44.4:11.3:1. (d) EDX mapping of Au, Ag and I, which are uniformly distributed. The scale bar is 200 nm. The area of elements mapping area is identical to that in the SEM images.

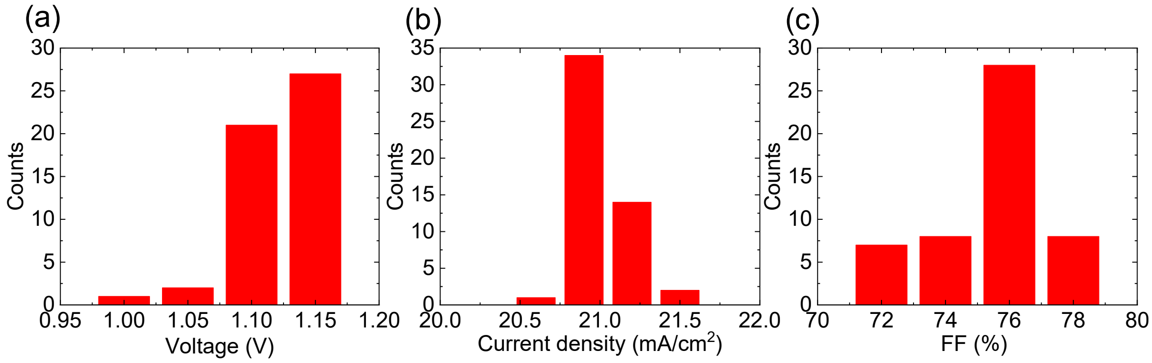

Figure S9. Statistical data of the photovoltaic performances of nano-Au/PSCs. (a–c) V_{OC}, J_{SC}, and FF of PSCs, respectively.

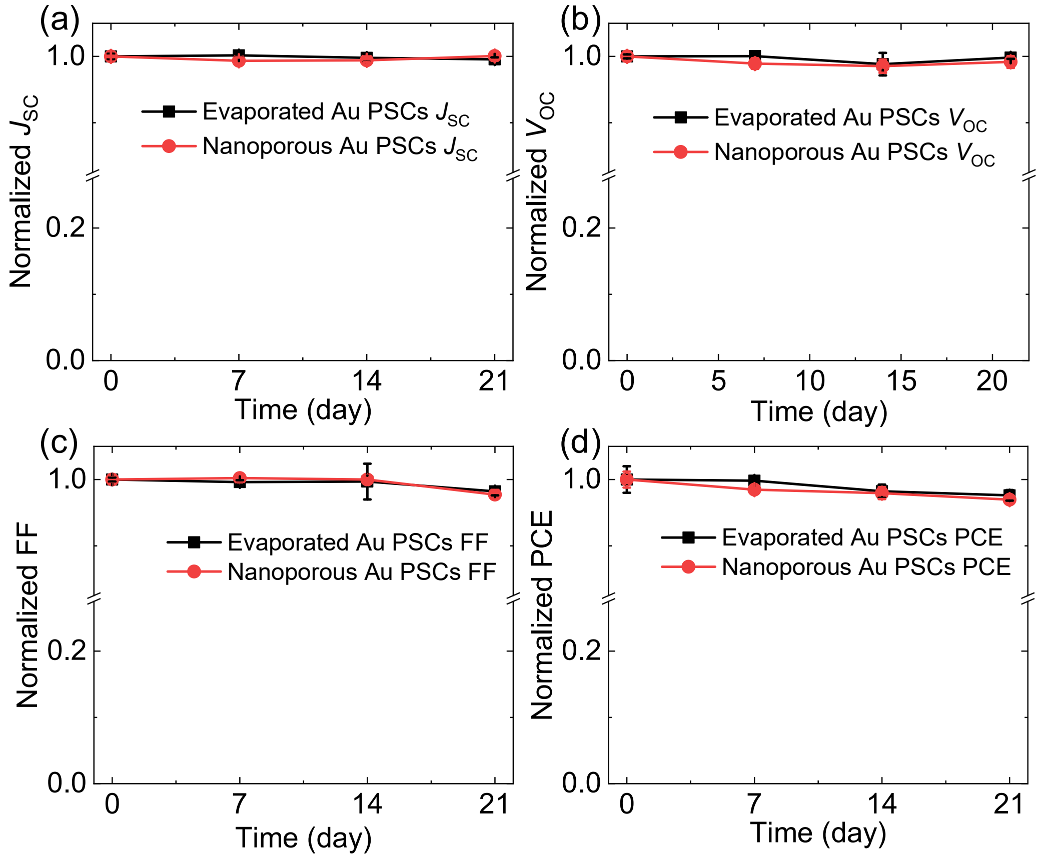

Figure S10. Storage stability characterization of the evaporated and nanoporous Au PSCs with three devices. (a–d) Normalized J_{SC}, V_{OC}, FF, and PCE of the evaporated and nanoporous Au PSCs, respectively. All devices were stored under ambient conditions at a humidity of ca. 25%.

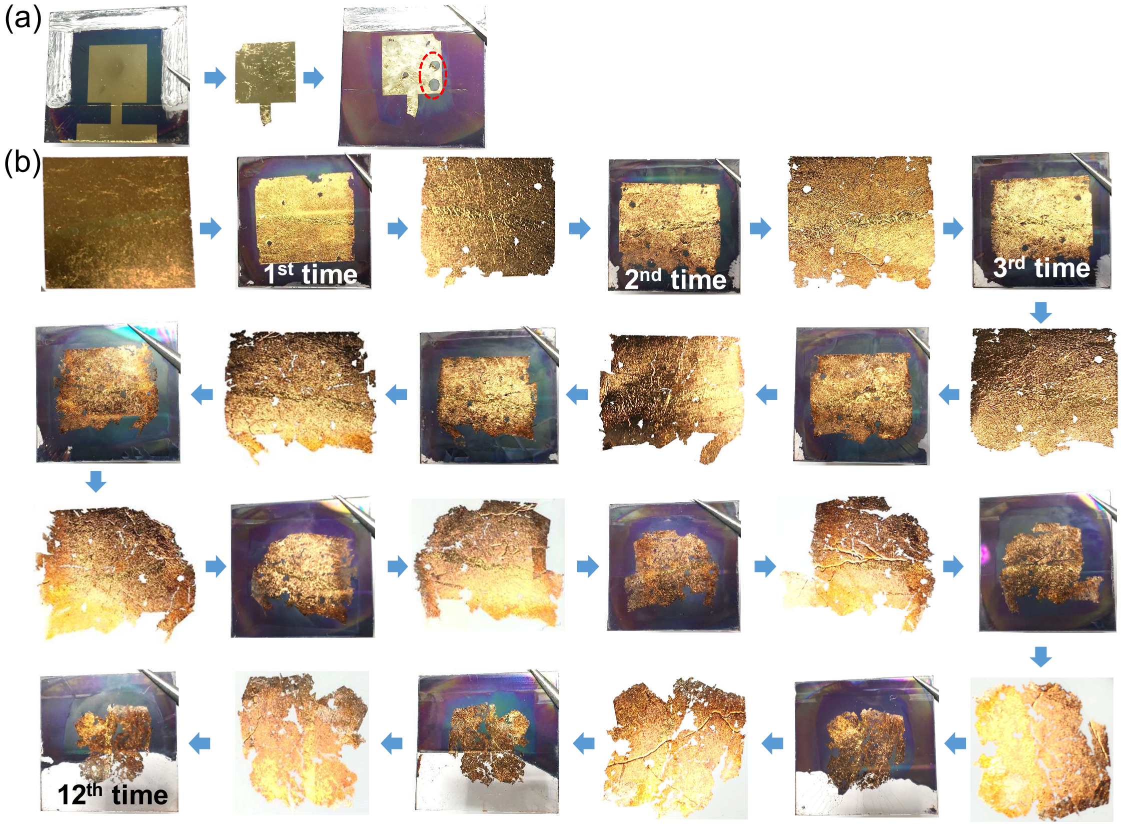

Figure S11. Photographs of Au films and PSCs during recycling. (a) Recycling of evaporated Au film and PSCs. (b) Recycling of nanoporous Au film and PSCs. The size of the PSC substrate is 25 × 25 mm^2.

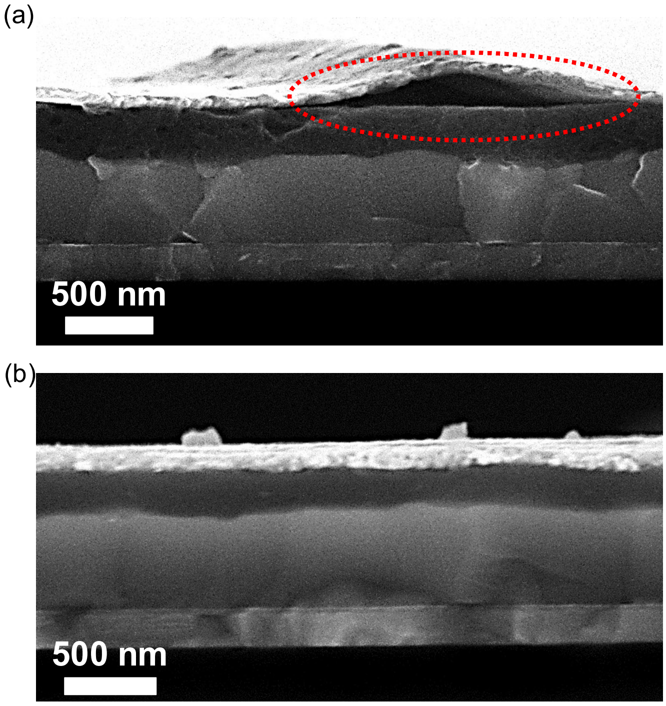

Figure S12. (a, b) Cross-sectional SEM images of PSCs with evaporated and nanoporous Au electrodes after recycling, respectively. The scale bar is 500 nm.

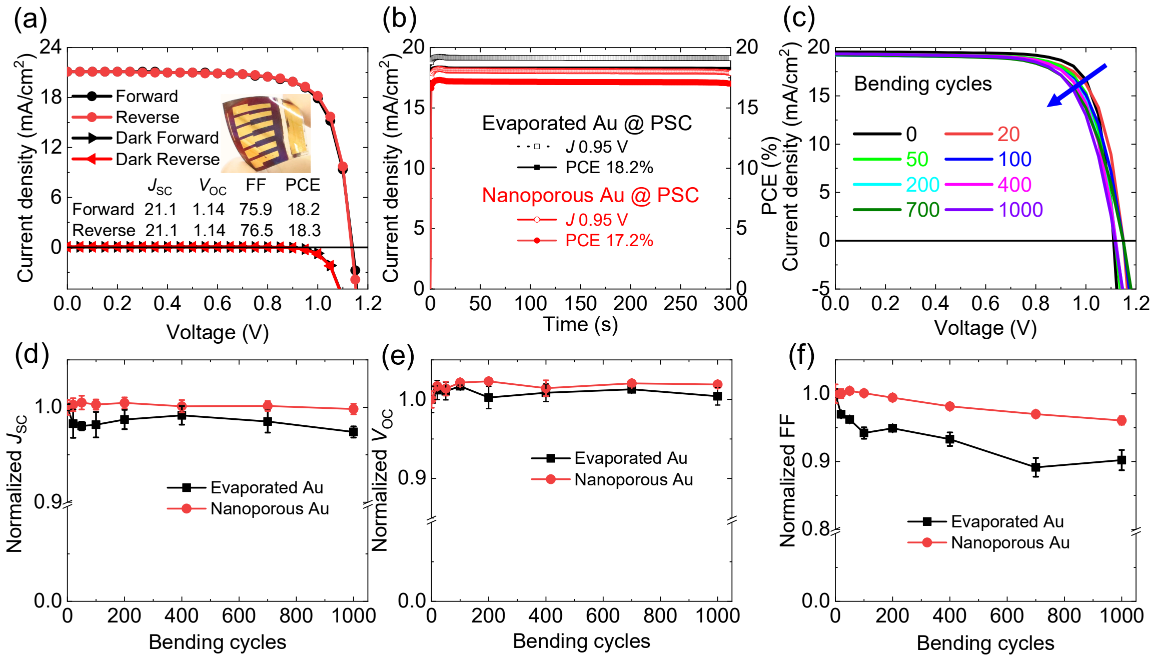

Figure S13. Photovoltaic performance of evap- and nano-Au/fPSCs. (a) *J–V* curves, (b) SPO of evap- and nano-Au/fPSCs. (c) *J–V* curves of evap-Au/fPSCs under various bending cycles. (d–f) Statistical summary of the bending durability: normalized J_{SC}, V_{OC} and FF of evap- and nano-Au/fPSCs, respectively.

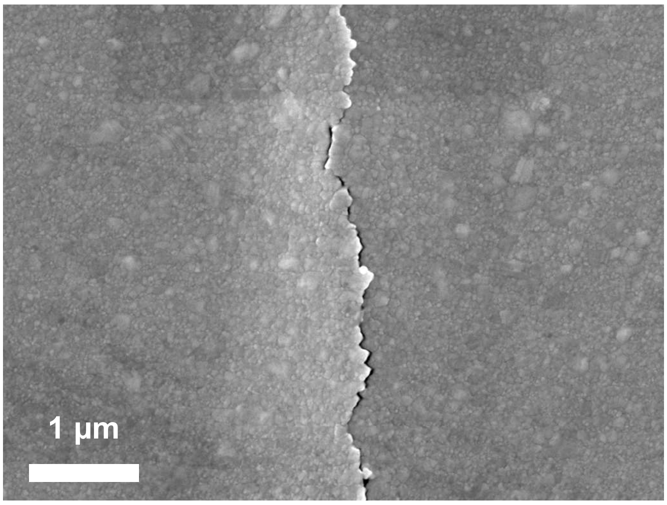

Figure S14. Surface morphology of evaporated Au film after 400 bending cycles at a bending radius of 5 mm. The scale bar is 1 μm of SEM image.

Table S1. Summary of photovoltaic performance of standard PSCs without PMMA and PSCs with the PMMA:spiro-OMeTAD layer based on various concentrations of spiro-OMeTAD (concentration ratio: *wt*/*wt*).

Concentration ratio	Scanning direction	J_{SC} (mA/cm^2)	V_{OC} (V)	FF (%)	PCE (%)
Standard PSCs	Forward	21.6	1.15	72.5	18.0
	Reverse	21.6	1.16	74.1	18.6
10:0.0 PSCs	Forward	21.9	1.16	73.2	18.5
	Reverse	22.0	1.16	77.3	19.7
10:0.5 PSCs	Forward	22.3	1.17	72.3	18.8
	Reverse	22.3	1.16	75.0	19.4
10:1.0 PSCs	Forward	22.4	1.17	74.5	19.5
	Reverse	22.4	1.17	75.9	19.8
10:1.5 PSCs	Forward	22.3	1.16	79.0	20.4
	Reverse	22.3	1.16	78.9	20.4
10:2.0 PSCs	Forward	21.5	1.16	72.2	17.9
	Reverse	21.5	1.15	74.6	18.6

Table S2. Summary of time-resolved PL decay of perovskite film without and with the PMMA:spiro-OMeTAD layer. The parameters of a_1, τ_1, τ_2, and τ_{PL} denote the amplitude ratio, lifetime constants of the fast and slow components, and the averaged PL decay based on two-exponential decay functions, respectively.

Sample	a_1	τ_1 (ns)	τ_2 (ns)	τ_{PL} (ns)
Standard pero	0.52 ± 0.01	25.7 ± 1.6	162.3 ± 6.9	92.2 ± 3.0
Passivated pero	0.46 ± 0.01	44.4 ± 2.4	177.7 ± 1.7	115.9 ± 2.7

Table S3. Summary of photovoltaic performance of PSCs with evaporated and nanoporous Au electrodes after recycling (once).

Sample	Reverse scan	J_{SC} (mA/cm^2)	V_{OC} (V)	FF (%)	PCE (%)
Evaporated Au PSCs	1st time	21.8	1.04	78.2	17.7
	2nd time	20.7	0.94	62.7	12.3
Nanoporous Au PSCs	1st time	21.6	0.97	79.1	16.5
	2nd time	21.6	0.97	79.0	16.5

Table S4. Calculated costs of chemicals used in each PSC using evaporated and nanoporous Au films as electrodes, including one, six, and 12 iterations of recycling. The volume of nanoporous Au film is estimated as 20 × 20 × 95 nm. The currency exchange ratio is 105.4 between the US dollar and Japanese yen.

Chemicals	Price (yen/g)	Company	1 piece cost (yen)	Evaporation device (yen)	Nanopsrous device (yen)
Au	5300	Tanaka Kikinzoku Kogyo	178.6	178.6	
Ag	60.37	Tanaka Kikinzoku Kogyo			
PbI_2	2200	TCI	223.1	223.1	223.1
$PbBr_2$	4600	TCI	67.5	67.5	67.5
CsI	500	TCI	1.3	1.3	1.3
FAI	6500	TCI	223.6	223.6	223.6
tBP	1660	TCI	16.5	16.5	16.5
Co-TFSI	780	Sigma-Aldrich	4.9	4.9	4.9
Li-TFSI	276	Wako	1.2	1.2	1.2
spiro-OMeTAD	7800	Merck	63.7	63.7	63.7
SnO_2	18	Alfa Aesar	1.9	1.9	1.9
ITO	135 (piece)	Geomatec	135	135	135
$Au_{35}Ag_{65}$ film			1.1		1.1
One time use				917.5 (8.71 $)	740.0 (7.03 $)
6 times use				5505 (52.27 $)	4433.4 (41.99 $)
12 times use				11010 (104.53 $)	8866.8 (83.61 $)

Table S5. Statistics of photovoltaic parameters of 10 evap-Au/fPSCs and 20 nanoporous Au/fPSCs.

Sample	Scanning direction	J_{SC} (mA/cm^2)	V_{OC} (V)	FF (%)	PCE (%)
Evaporated Au flexible PSCs	Forward	20.61 ± 0.49	1.13 ± 0.01	75.7 ± 0.0	17.56 ± 0.48
	Reverse	20.65 ± 0.49	1.12 ± 0.01	76.6 ± 0.0	17.73 ± 0.49
Nanoporous Au flexible PSCs	Forward	19.47 ± 0.15	1.12 ± 0.01	75.5 ± 0.0	16.45 ± 0.36
	Reverse	19.50 ± 0.15	1.12 ± 0.01	76.2 ± 0.0	16.65 ± 0.41

Reference

[1] F. Yang, J. Liu, H. E. Lim, Y. Ishikura, K. Shinokita, Y. Miyauchi, A. Wakamiya, Y. Murata, K. Matsuda, *J. Phys. Chem. C* **2018**, *122*, 17088.

[2] R. Liquid, J. Erlebacher, M. J. Aziz, A. Karma, N. Dimitrov, *Nature* **2001**, *410*, 5.

[3] L. Y. Chen, Y. Hou, J. L. Kang, A. Hirata, T. Fujita, M. W. Chen, *Adv. Energy Mater.* **2013**, *3*, 851.

[4] X. Ge, L. Chen, L. Zhang, Y. Wen, A. Hirata, M. Chen, *Adv. Mater.* **2014**, *26*, 3100.

[5] L. Y. Chen, X. W. Guo, J. H. Han, P. Liu, X. D. Xu, A. Hirata, M. W. Chen, *J. Mater. Chem. A* **2015**, 3, 3620.

[6] F. Yang, J. Liu, X. Wang, K. Tanaka, K. Shinokita, Y. Miyauchi, A. Wakamiya, K. Matsuda, *ACS Appl. Mater. Interfaces* **2019**, *11*, 15680.

[7] F. Yang, H. E. Lim, F. Wang, M. Ozaki, A. Shimazaki, J. Liu, N. B. Mohamed, K. Shinokita, Y. Miyauchi, A. Wakamiya, Y. Murata, K. Matsuda, *Adv. Mater. Interfaces* **2018**, *5*, 1701256.
